# Exploring the Role of microRNAs as Blood Biomarkers in Alzheimer’s Disease and Frontotemporal Dementia

**DOI:** 10.3390/ijms26073399

**Published:** 2025-04-05

**Authors:** Irene Petracci, Sonia Bellini, Katarzyna Goljanek-Whysall, Leo R. Quinlan, Agnieszka Fiszer, Ali Cakmak, Cyrille Mesue Njume, Barbara Borroni, Roberta Ghidoni

**Affiliations:** 1 Molecular Markers Laboratory, IRCCS Istituto Centro San Giovanni di Dio Fatebenefratelli, 25125 Brescia, Italy; ipetracci@fatebenefratelli.eu (I.P.); sbellini@fatebenefratelli.eu (S.B.); bborroni@fatebenefratelli.eu (B.B.); 2 Discipline of Physiology, School of Medicine, University of Galway, H91 TH33 Galway, Irelandleo.quinlan@universityofgalway.ie (L.R.Q.); 3 Institute of Life Course and Medical Sciences (ILCAMS), University of Liverpool, L7 8TX Liverpool, UK; 4 Galway RNA Research Cluster, University of Galway, H91 TK33 Galway, Ireland; 5 Department of Medical Biotechnology, Institute of Bioorganic Chemistry, Polish Academy of Sciences, Noskowskiego 12/14, 61-704 Poznan, Poland; agnieszka.fiszer@ibch.poznan.pl; 6 Department of Computer Engineering, Ayazaga Campus, Istanbul Technical University, Reşitpaşa, Sarıyer, 34467 Istanbul, Turkey; ali.cakmak@itu.edu.tr (A.C.); cyrillemesue@gmail.com (C.M.N.); 7 Department of Clinical and Experimental Sciences, University of Brescia, 25123 Brescia, Italy

**Keywords:** miRNAs, biomarkers, extracellular vesicles, Alzheimer’s disease, frontotemporal dementia

## Abstract

Alzheimer’s disease (AD) and frontotemporal dementia (FTD) are the most common forms of dementia globally. AD is characterized by the accumulation of amyloid-β (Aβ) plaques and hyperphosphorylated tau in the brain, leading to progressive memory loss and cognitive decline, significantly impairing daily life. In contrast, FTD is marked by selective degeneration of the frontal and/or temporal lobes, typically resulting in profound changes in personality and social behavior, speech disorders, and psychiatric symptoms. Numerous studies have found microRNAs (miRNAs)—small, non-coding RNA molecules that regulate gene expression post-transcriptionally—to be dysregulated in AD and FTD. As a result, miRNAs have emerged as promising novel biomarkers for these diseases. This review examines the current understanding of miRNAs in AD and FTD, emphasizing their potential as accessible, noninvasive biomarkers for diagnosing these prevalent neurodegenerative disorders.

## 1. Introduction

Dementia is a clinical syndrome characterized by the progressive loss of cognitive functioning (memory, thinking, and reasoning) and emotional abilities to the point where daily life and activities are significantly impaired. Alzheimer’s disease (AD) and frontotemporal dementia (FTD) are the most prevalent forms of dementia worldwide [1]. AD is characterized by the neuropathological accumulation of protein aggregates (hyperphosphorylated tau, neurofibrillary tangles, and extracellular Aβ plaques) and synaptic loss in the medial temporal lobe and neocortex. At the same time, FTD encompasses a heterogeneous group of progressive brain disorders characterized by atrophy of the prefrontal and anterior temporal lobes [2]. While genetic mutations are known to contribute to the familial nature of these diseases, a significant number of AD and FTD cases are sporadic. These cases arise from a complex interplay of age-related brain changes and genetic, environmental, and lifestyle factors. Neuroinflammation and oxidative damage are well established as central players in neurodegeneration. The brain is particularly vulnerable to oxidative stress due to its high oxygen consumption (resulting from substantial ATP demand), its abundance of polyunsaturated fatty acids, the accumulation of redox-active metal ions, relatively low antioxidant defenses, and the predominance of non-mitotic cells [3]. As a result, the unchecked generation of reactive oxygen species (ROS), which may occur due to impaired mitochondrial function or reduced ROS buffering capacity, damages lipids, proteins, and nucleic acids in the brain. This damage initiates a feedback loop that further increases the susceptibility of neural tissues to damage [4,5,6,7,8,9,10]. Additionally, oxidative cross-linking makes proteins more resistant to proteolytic degradation by the proteasome system, promoting the accumulation of abnormal protein aggregates, a hallmark of neurodegenerative disorders [11]. Currently, there is no established strategy for preventing AD and FTD, and therapeutic options remain limited due to the complex nature of these diseases. Diagnosis typically relies on symptomatic presentation, often after significant neuronal damage has already occurred. Given the millions of people worldwide living with AD and FTD, along with the devastating impact on patients, families, and healthcare systems, identifying strategies to prevent, slow, and potentially cure these conditions is a critical priority for scientists globally. Over the past decade, significant progress has been made in researching biomarkers for neurodegenerative diseases, which could facilitate early detection, monitor disease progression, and serve as targets for novel therapies. Notably, recent studies have identified dysregulated microRNAs (miRNAs) in circulating extracellular vesicles (EVs) and plasma/serum as common features in both AD and FTD patients [12,13]. These findings suggest that miRNAs could be potential biomarkers for these diseases. This review aims to provide a comprehensive overview of the current understanding of the role of miRNAs in AD and FTD, highlighting their potential use as accessible, noninvasive biomarkers for diagnosing these two prevalent neurodegenerative disorders.

## 2. miRNA Biogenesis

**Figure 1:**
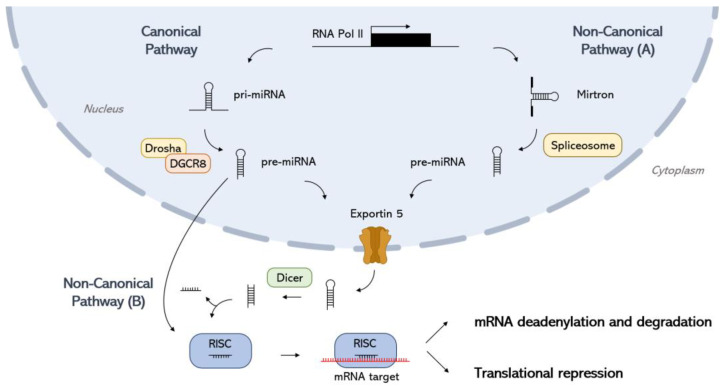
Canonical and non-canonical miRNA biogenesis pathways. Canonical pathway: RNA polymerase II (RNA Pol II) transcribes the miRNA gene to form a primary miRNA (pri-miRNA). Pri-miRNA is then processed by Drosha and the RNA-binding protein DiGeorge Syndrome Critical Region 8 (DGCR8), producing a precursor miRNA (pre-miRNA). The pre-miRNA is transported to the cytoplasm by Exportin-5 and is cleaved by Dicer to form a duplex mature miRNA. The mature miRNA directs the Ago protein complex (the miRNA-induced silencing complex, or the RISC) to mRNA cleavage and degradation or translational repression. Non-canonical pathway (A)—the synthesis of miRNA by the Drosha/DGCR8 independent pathway: primary miRNA is spliced by a spliceosome, forming a branched pre-miRNA. The pre-miRNA is debranched by a debranching enzyme, after which the synthesis is similar to the canonical pathway. Non-canonical pathway (B)—the synthesis of miRNA by the Dicer-independent pathway: the pre-miRNA formed through the cleavage of pri-miRNA by Drosha/DGCR8 is exported to the cytoplasm. The pre-miRNA is not long enough to be processed by Dicer and forms the RISC.

miRNAs were first discovered in 1993 in Caenorhabditis elegans by the Ambros and Ruvkun groups, marking the beginning of regulatory research in molecular biology [14,15]. miRNAs are a class of small non-coding RNAs, typically 18–22 nucleotides long, that play crucial roles in gene expression regulation through RNA interference. By binding to specific target mRNAs, miRNAs either promote their degradation or inhibit translation. Highly conserved across animal species, miRNAs are essential for normal development and cellular processes such as proliferation, differentiation, and apoptosis [16]. Disruptions in miRNA expression are associated with various health conditions, including cancer, bone disease, cardiovascular disease, autoimmune disorders, viral infections, and neurological diseases. Since their discovery, advancements in experimental techniques have underscored the significant role of miRNAs in both development and disease, transforming the field of molecular biology. miRNAs are derived from intergenic or intragenic regions of the genome, transcribed from introns or non-coding RNA (ncRNA) genes. Approximately 40% of human miRNAs are found in clusters, predominantly from intergenic regions, where they are regulated by promoters and transcribed independently [17,18,19]. These miRNA clusters can be transcribed as polycistronic transcripts that encode multiple miRNAs, which may share similar expression patterns and functions, though not always identical. Multiple mechanisms regulate miRNA biogenesis, generally classified into canonical and non-canonical pathways (Figure 1). In the canonical pathway, which is predominant, miRNA genes are transcribed by RNA polymerase II into primary miRNAs (pri-miRNAs) that contain double-stranded, hairpin-like structures. These pri-miRNAs are processed in the nucleus by the “microprocessor” complex, composed of the ribonuclease III enzyme Drosha and the RNA-binding protein DiGeorge Syndrome Critical Region 8 (DGCR8) [20]. DGCR8 recognizes an N6-methyladenylated GGAC and other motifs within the pri-miRNA, while Drosha cleaves the pri-miRNA at the base of the hairpin stem [21,22], releasing a precursor miRNA (pre-miRNA) of about 55–70 nucleotides with a two-nucleotide 3′ overhang. The pre-miRNA is exported to the cytoplasm by the nuclear transport receptor, Exportin-5 (Exp5), where it is further processed into a miRNA duplex (21–22 nucleotides long) by Dicer, which removes the terminal loop. A peculiar feature of the RNA duplex is the presence of bulges and base mismatches, which cause imperfect strand pairing. The duplex is loaded onto an Argonaute (Ago) protein, and after the passenger strand is discarded, the mature miRNA directs the Ago protein complex (the miRNA-induced silencing complex, or the RISC) to silence complementary mRNA targets via sequence complementarity to the 3′ untranslated region (3′ UTR) of the target mRNA [23]. Several non-canonical miRNA biogenesis pathways also exist, bypassing some canonical pathway components. These include Drosha/DGCR8-independent or Dicer-independent pathways [24]. For example, mirtrons are a class of introns that bypass the Drosha/DGCR8 complex. Instead, they are processed by spliceosomes and debranching enzymes in the nucleus to form miRNA hairpins. These hairpins are then exported from the nucleus by Exportin-5 (Exp5) and undergo further processing by Dicer and the RISC in the cytoplasm. Conversely, short hairpin RNAs (shRNAs) are processed by Drosha in the nucleus but do not require Dicer for further maturation in the cytoplasm [25]. The main components of the RISC complex are Dicer, the transactivation response element RNA-binding protein (TRBP), and Argonaute2 (Ago2), which serves as the catalytic center of the RISC. Ago2 is essential for strand selection and coordinating target gene silencing events [26]. Both strands of the pre-miRNA (“5p” and “3p”) can be functional, each possessing a unique seed sequence that is complementary to a target mRNA, thus allowing their selection during RISC loading [27]. The strand loaded into Ago and becoming part of the RISC is referred to as the “guide” strand, while the other strand, known as the “passenger” strand, is discarded and degraded. Strand selection is influenced by two main factors: the identity of the 5′ nucleotide on each strand and the relative thermodynamic stability of the duplex ends [27]. Ago2 shows a stronger binding affinity for the 5′ mono-phosphorylated uracil on miRNA strands and preferentially loads the strand with the lower thermodynamic stability. A single hydrogen bond difference can determine which strand is loaded in vitro [28,29,30,31,32].

## 3. miRNA-Mediated Gene Regulation

In animals, miRNA-mediated gene regulation occurs post-transcriptionally and is a highly dynamic process. Within the RISC, miRNAs guide Ago proteins to complementary mRNA targets, either silenced or degraded. The guide strand loaded onto the RISC determines the specific mRNA target. The first 2–8 nucleotides from the 5′ end of the guide strand, known as the “seed region”, are primarily responsible for base pairing, as they recognize the miRNA response element (MRE), typically located in the 3′ untranslated region (3′ UTR) of the target mRNA. Additionally, nucleotides in the 3′ half of the miRNA can participate in further base pairing with the target mRNA. miRNAs silence gene expression by repressing translation and/or promoting mRNA decay through deadenylation, decapping, and subsequent exonucleolytic digestion [33]. The effectiveness of miRNA-mediated gene silencing depends on the complementarity between the seed region and the MRE. A fully complementary miRNA–MRE interaction activates Ago2 endonuclease activity, leading to mRNA cleavage and degradation. However, most miRNA–MRE interactions in animal cells are not entirely complementary, often due to central mismatches in the binding site, which prevent Ago2-mediated cleavage and result in translation repression [34]. Several studies have reported that miRNAs can upregulate gene expression under certain conditions [35,36].

Furthermore, miRNA-mediated regulation is not uniform across all cell types. The response to miRNA regulation can vary depending on the availability of MREs in a cell-type or state-specific manner [24]. The RISC and target mRNA have been observed in various subcellular compartments, including the rough endoplasmic reticulum, processing (P)-bodies, the trans-Golgi network, early/late endosomes, multivesicular bodies, lysosomes, mitochondria, and the nucleus [37,38,39,40]. In addition to canonical post-transcriptional regulation, a novel role for miRNAs as extracellular signaling molecules has recently been suggested, particularly in the activation of the Toll-like receptor (TLR) family. For example, tumor-secreted miR-21 and miR-29a can act as ligands for the murine TLR7 and human TLR8 receptors on immune cells, triggering a TLR-mediated prometastatic inflammatory response that may ultimately lead to tumor growth and metastasis [41]. Interestingly, let-7, a highly abundant regulator of gene expression in the central nervous system (CNS), which is found in elevated amounts in the cerebrospinal fluid (CSF) of individuals with AD, has also been shown to activate TLR7 signaling in macrophages and microglia, exerting neurotoxic effects in cortical and hippocampal neurons [42]. Similarly, miR-100-5p and miR-298-5p, acting as endogenous ligands for TLR7/8 in the CNS, induce an inflammatory response through the activation of microglia and macrophages [43]. Additionally, miRNAs can be released into extracellular fluids, reaching distant target cells and exerting hormone-like effects, acting as autocrine, paracrine, and/or endocrine regulators of cellular functions [44,45,46]. Due to their stability in body fluids, extracellular miRNAs are actively being studied as potential biomarkers for various human diseases.

## 4. Extracellular miRNAs and Circulating EVs

The discovery of cell-free miRNAs dates to 2008, when Chim et al. first identified placental miRNAs in maternal plasma [47]. Since then, cell-free miRNAs have been detected in various biological fluids, including cerebrospinal fluid, saliva, breast milk, urine, amniotic fluid, and tears [48,49,50,51,52,53]. The concentration and composition of these miRNAs can vary significantly depending on the type of fluid [53]. The population of extracellular miRNAs within biological fluids is highly heterogeneous. In blood, most circulating miRNAs (90–95%) are associated with proteins, primarily Ago2, high-density lipoprotein (HDL), and nucleophosmin 1, which protect miRNAs from nuclease degradation [49,54,55,56]. These circulating miRNAs likely originate from the passive release of cytosolic components during cell death. However, some studies suggest that miRNAs in serum and saliva are primarily enclosed within EVs [51]. Circulating EVs, such as exosomes, microvesicles, and apoptotic bodies, resist RNase activity, further protecting miRNAs from degradation [45,51]. EVs are a diverse group of nano- to micrometer-sized, membrane-bound lipid vesicles released by all eukaryotic cells. They are abundant in almost all human body fluids and serve various biological functions, particularly in intracellular communication, immune response, and antigen presentation. EVs are classified based on their biogenesis, with the most common types being microvesicles, exosomes, and apoptotic bodies [57,58]. Apoptotic bodies (0.05–3 μm) are subcellular vesicles released during apoptosis, or programmed cell death, through a process known as apoptotic cell disassembly [59]. Microvesicles (100 nm–1 μm), also called shedding vesicles, are generated by the outward budding of membrane vesicles from the plasma membrane, followed by separation [60,61]. Lastly, exosomes (30–100 nm) are much smaller membrane particles of endosomal origin. They form intracellularly through the inward invagination of endosomal membranes to create multivesicular bodies (MVBs), which are released into the extracellular space upon fusion with the plasma membrane [62,63]. The primary function of EVs is to transport bioactive molecules, or “cargo”, such as lipids, sugars, proteins, and nucleic acids (including non-coding RNAs like miRNAs) to recipient cells.

Within EVs, the cargo is shielded by a lipid bilayer, providing protection from degradation and allowing fragile molecules like miRNAs to remain stable. Although the precise mechanisms by which miRNAs are selectively packaged into EVs remain unclear, the process is believed to be highly selective [64]. The content of EVs varies depending on the cell of origin and its physiological state. In pathological conditions, the EV formation process can be disrupted, leading to abnormal shedding and altered levels of EVs in bodily fluids. Several studies have shown that the miRNA signature associated with EVs reflects molecular changes in the cells from which they originate. These miRNA profiles can be characteristic of the disease stage, offering significant diagnostic value [65,66]. Therefore, EVs and their cargo are being explored as potential biomarkers and tools for future diagnostic and therapeutic strategies.

## 5. Crosstalk Between miRNAs and Oxidative Stress Pathways

Neuroinflammation plays a central role in the initiation and progression of neurological disorders. While low levels of ROS are important for healthy aging, as they contribute to physiological processes like immunity and metabolism, excessive ROS production alters the cellular redox state, leading to oxidative stress [67] and disrupting cell and tissue homeostasis. Excess ROS can damage lipids, proteins, and nucleic acids, leading to a progressive decline in physiological functions. This damage is a key contributor to aging and the development of various pathologies, including cancer, cardiovascular diseases, diabetes, and neurological disorders. Lipid peroxidation, which occurs when ROS damage fatty acids, leads to the degradation of lipids and cell membrane damage. Proteins can be reversibly or irreversibly altered by oxidative stress, resulting in either the loss of function or protein aggregation [68]. Similarly, both RNA and DNA are vulnerable to oxidative damage. Guanine (G) is the nucleic acid base most susceptible to ROS damage due to its low redox potential [69]. The oxidative modification of guanine, known as 8-oxoguanine, is a key biomarker of oxidative stress in DNA and RNA. This modification can occur in DNA (8-oxo-dG), RNA (8-oxo-G), or in the free nucleotide form (8-oxo-dGTP or 8-oxo-GTP), which can then be incorporated during DNA replication or RNA transcription. Oxidized guanine can be detected using high-performance liquid chromatography, immunoblotting, and mass spectrometry [70]. In humans, RNA is more prone to oxidative damage than DNA, likely due to its inherent instability (due to the reactive 2′-hydroxyl group), single-stranded nature, lack of protein protection, absence of redundant repair systems, and proximity to ROS production sites in the cell [71]. However, RNA oxidative damage has received relatively little attention [72]. Recent research has highlighted a significant connection between oxidative stress and non-coding RNAs, especially miRNAs.

Oxidative stress can lead to aberrant miRNA expression [73], and specific miRNA subsets may be released from exosomes during pro-inflammatory or oxidative stress conditions. Multiple miRNAs are thought to regulate genes involved in mitochondrial integrity and oxidative stress [74]. Consequently, altered miRNA levels have been linked to mitochondrial dysfunction and impaired ROS production in several neurodegenerative diseases, including AD, Parkinson’s disease (PD), and Huntington’s disease [75,76]. Moreover, miRNAs themselves can be direct targets of ROS-induced oxidation. Oxi-miRs can still be associated with the RISC complex [73]. Still, since oxidized guanine (oxo-G) pairs preferentially with adenine instead of cytosine, this modified base may redirect the miRNA’s target recognition. This alteration, particularly within the seed region of miRNAs, can target non-native mRNAs and cause profound changes in gene expression [73,77]. Although this phenomenon has not been fully explored, recent studies suggest that oxo-G in miRNAs can significantly disrupt their regulatory function. Interestingly, miRNA oxidation appears selective, as not all miRNAs are oxidized in response to oxidative stress, and the process does not seem to correlate with miRNA abundance. However, whether some specific motifs or sequences make certain miRNAs more prone to oxidative modification or whether cofactors mediate this process remains unclear. Oxi-miRs have been implicated in various diseases. For example, studies on rat heart cell lines (H9c2) and mouse models have shown that oxidative modification of miR-184 promotes association with Bcl-xL and Bcl-w, leading to apoptosis and contributing to cardiac infarction [77]. Similarly, Seok and colleagues demonstrated that oxidation at specific positions in the seed region of miR-1b induces phenotypic changes characteristic of cardiac hypertrophy in the same cell lines and models [73]. In addition, RNA oxidation has been demonstrated in AD [71,78,79], further supporting the importance of oxidized RNA, including miRNAs, in neurodegeneration.

## 6. miRNAs as Potential Biomarkers in AD

In humans, miRNAs play a crucial role in post-transcriptional gene regulation, influencing the expression of up to 60% of protein-coding genes [80]. By modulating the expression of key genes and cellular pathways, miRNAs can contribute to the development of various diseases [81]. Dysregulation of circulating miRNAs with EVs that carry them has emerged as a potential contributor to neurodegeneration, positioning miRNAs as promising biomarkers for early disease detection. In AD, miRNA dysregulation alters cellular mechanisms involved in AD pathogenesis. For instance, miR-101, miR-20a, miR-17-5p, and miR-106b negatively regulate amyloid precursor protein (APP) [82,83], from which Aβ is produced via β-site APP-cleaving enzyme 1 (BACE1)-mediated cleavage. Similarly, miR-149, miR-34a-5p, miR-125b-5p, miR-15b, miR-16, miR-124, and miR-374b-5p target BACE1 to modulate amyloid-β production [84,85,86,87,88,89]. Instead, miR-132, one of the most abundant and brain-enriched miRNAs, is consistently downregulated in the brains of individuals with AD [90]. This downregulation affects various physiological processes, including neuronal differentiation, neurite outgrowth, synaptic plasticity, neuronal apoptosis, and survival [90]. Regarding tau protein, miR-132 and miR-125b promote tau phosphorylation [91,92], while miR-483–5p, miR-125b-5p, and miR-23b indirectly regulate tau phosphorylation through the extracellular signal-regulated kinases 1 and 2 (ERK1/2), protein phosphatase methylesterase-1 (PME-1), and N-acetylglucosaminyltransferase III (GNT-III) signaling pathways [93,94,95]. Additionally, miR-16–5p and miR-195 can disrupt mitochondrial membrane potential and integrity [96,97], while miR-210, miR-338, and miR-34a inhibit respiratory enzyme activity, leading to energy deficits in neurons and exacerbating AD progression [98,99,100]. miRNA profiles in AD patients’ brains and peripheral blood often differ from those in healthy individuals. These changes may be stage- or region-specific, making them potential biomarkers for early cognitive decline detection. Although several studies have investigated miRNA biomarkers for AD, a clear and consistent differential expression pattern has not yet emerged. This inconsistency is largely due to differing sample matrices (e.g., blood, brain, and cerebrospinal fluid), methodological variations, and small sample sizes, which make cross-study comparisons and replication across independent cohorts challenging. As a result, no consensus has been reached regarding specific miRNAs that are differentially regulated in AD versus healthy controls. Despite these challenges, a panel of multiple miRNAs could potentially aid in diagnosing AD at various disease stages. For example, Siedlecki-Wullich et al. investigated plasma miRNA levels in healthy controls, mild cognitive impairment (MCI) patients, and patients with AD or FTD. They found significant upregulation of miR-92a-3p, miR-181c-5p, and miR-210-3p in the plasma of both MCI and AD patients but not in FTD patients. These findings suggest the possibility of a miRNA signature specific to AD [101]. In a recent study, Wu et al. conducted cross-sectional comparisons and identified 71 miRNAs significantly differentially expressed between AD and control groups. Among these, two miRNAs—blood miR-146b-5p and miR-15b-5p—showed consistent downregulation across both the AD and control groups, even in an independent cohort [102]. The downregulation of the miRNA-15b concentration was also reported by Vergallo et al., who measured significantly lower plasma levels of miRNA-15b in Aβ-PET-positive compared to Aβ-PET-negative individuals [103]. Bioinformatic analysis revealed that miR-15b-5p targets mRNAs associated with cell cycle regulation and apoptosis. At the same time, miR-146b-5p plays a role in the innate immune response and cytokine production via the Toll-like receptor signaling pathway. In another study, Tan et al. used integrated statistical and machine learning approaches to identify three miRNAs that were differentially downregulated in AD: hsa-miR-6501-5p, hsa-miR-4433b-5p, and hsa-miR-143-3p [104]. The association of hsa-miR-4433b-5p with neurodegenerative disorders, including AD, PD, and FTD, has been previously documented, likely due to its role in regulating glial cells and the neuroimmune system [105,106]. The downregulation of hsa-miR-143-3p, also suggested as a potential AD biomarker in other reviews, is believed to contribute to AD by promoting tau phosphorylation and increasing APP levels, leading to Aβ accumulation [107]. While Tan et al.’s findings align with the literature, the study’s small sample size remains a limitation, and further validation is needed. Swarbrick et al. conducted a systematic review to quantify miRNAs that were significantly deregulated in the peripheral blood of AD patients, cross-referencing them with miRNAs known to be deregulated in limbic regions of the brain across different stages of AD. The review identified 10 miRNAs (miR-26b, miR-30e, miR-34a, miR-34c, miR-107, miR-125b, miR-146a, miR-200c, miR-210, and hsa-miRNA-485) that showed deregulation in AD, with changes detectable up to 20 years before the onset of clinical symptoms [108]. In a comprehensive meta-analysis, Yoon et al. screened 2744 articles, selecting 61 studies that included 512 miRNAs for analysis. The study highlighted several miRNAs differentially expressed in AD patients’ blood compared to healthy controls [109]. Notably, miR-30e-5p, miR-18b-5p, miR-424-5p, miR-582-5p, miR-335-5p, miR-20a-5p, miR-106a-5p, miR-361-5p, and miR-15a-5p were significantly upregulated, while miR-29b-3p, miR-27b-3p, miR-221-3p, miR-146a-5p, miR-15b-3p, miR-31-5p, miR-9-5p, miR-107, miR-103a-3p, and miR-1306-5p were significantly downregulated in AD patients. These miRNAs are involved in critical cellular processes such as amyloid and tau processing, apoptosis, cell proliferation, immune response, and inflammation. miRNAs that regulate multiple pathways implicated in AD pathogenesis could serve as valuable therapeutic targets. Among these, miR-15a-5p, miR-103a-3p, miR-29a-3p, and miR-107 are particularly interesting. In particular, miR-107, which regulates BACE1, consistently showed alterations across studies, highlighting its potential as a biomarker. On the other hand, miR-103, which promotes neurite outgrowth and suppresses neuronal apoptosis by targeting prostaglandin-endoperoxide synthase 2 (PTGS2), exhibited some heterogeneity across studies. Several studies have reported dysregulation of miRNA content in EVs in neurodegenerative diseases, including AD, further expanding the list of potential miRNA biomarkers. Gamez-Valero et al. found that the levels of miR-451a, miR-21-5p, miR-23a-3p, miR-126-3p, let-7i-5p, and miR-151a-3p were significantly reduced in plasma-derived EVs from AD patients compared to healthy controls [110]. Similarly, another study observed that miR-424-5p, miR-93-5p, miR-1306-5p, and miR-3065-5p were lower in plasma-derived EVs from AD patients than in those from patients with other forms of dementia [111]. Additionally, miR-106a-5p, miR-16-5p, miR-17-5p, miR-195-5p, miR-19b-3p, miR-20a-5p, miR-223-3p, miR-25-3p, miR-296-5p, miR-30b-5p, miR-532-3p, miR-92a-3p, and miR-451a were found to be upregulated in plasma EVs from AD patients, with many of them already being linked to neurological diseases [112]. The ability to isolate cell type-specific EVs from systemic biofluids has been explored in numerous studies as a potential diagnostic tool for various diseases, including AD and FTD. For instance, Serpente et al. found that miR-23a-3p, miR-223-3p, and miR-190a-5p levels were significantly upregulated in neural-derived small EVs (NDEVs) from AD patients, while miR-100-3p was significantly downregulated compared to controls [113]. Kumar et al. analyzed miRNA expression levels in various brain cell-derived EV subtypes (from neurons, astrocytes, microglia, oligodendrocytes, pericytes, and endothelial cells) isolated from plasma samples of individuals with normal cognition (CN), MCI, MCI converting to AD (MCI-AD), and AD dementia. They found that miRNAs in these EV subtypes were differentially expressed in MCI, MCI-AD, and AD dementia groups compared to the CN group. Specifically, in AD, the researchers observed a significant upregulation of miR-106b-5p, miR-107, and miR-135b-5p in neuronal EVs from AD patients compared to controls. In contrast, miR-29a-5p, miR-9-5p, miR-125b-5p, miR-132-5p, and miR-210-3p exhibited variable expression levels, with either upregulation or downregulation in neuronal EVs from AD patients. Furthermore, the miRNA profiles in these EV subtypes were able to discriminate dementia status with an area under the curve (AUC) > 0.90, correlating with the thickness of the temporal cortical region [114]. Although these studies (Table 1) do not provide a comprehensive list of all dysregulated miRNAs in EVs, they underscore the potential of specific miRNAs in plasma-derived EVs as biomarkers for AD diagnosis and monitoring.

## 7. Computational Approaches to Discover miRNA Biomarkers for AD

Several computational methods have been developed to identify miRNAs that could serve as biomarkers for AD. These methods typically involve analyzing large datasets of miRNA expression data to identify miRNAs that are differentially expressed in AD patients compared to healthy controls. Table 2 summarizes some of the methods along with their proposed miRNA biomarkers. We next discuss different approaches to computational miRNA biomarker discovery under five umbrella categories.

### 7.1. Differential Expression Analysis of miRNAs in AD

Differential expression analysis involves comparing the expression levels of miRNAs in AD patients and healthy controls to identify miRNAs that are significantly upregulated or downregulated in AD. Differential expression analysis is a widely used approach for uncovering potential biomarkers and gaining insights into disease mechanisms. By identifying these alterations in miRNA expression levels, researchers can better understand the molecular changes associated with AD. Several studies have demonstrated that miRNA dysregulation plays a crucial role in AD pathogenesis, influencing key pathways such as neuroinflammation, Aβ metabolism, and synaptic dysfunction [104,115,116]. These findings suggest that differentially expressed miRNAs could serve as valuable biomarkers for early detection and as targets for therapeutic intervention. Various computational and statistical techniques are employed to perform differential expression analysis. High-throughput sequencing methods such as RNA sequencing (RNA-seq) and microarray technologies allow comprehensive profiling of miRNA expression across different biological samples [128]. RNA-seq offers higher sensitivity and dynamic range compared to microarrays, making it a preferred method for identifying low-abundance miRNAs [129]. To analyze sequencing data, statistical methods such as edgeR and DESeq2 are commonly used. These methods apply negative binomial models to account for variability and detect significant expression differences [130]. For microarray-based studies and RNA-seq data processed with the voom transformation, Limma is widely used, leveraging linear models and empirical Bayes moderation to improve variance estimation, particularly in studies with small sample sizes [131]. In addition to conventional statistical techniques, machine learning (ML) approaches are increasingly being integrated into differential expression analysis. Hybrid frameworks combining support vector machine recursive feature elimination (SVM-RFE) and conventional bioinformatics pipelines have been shown to enhance feature selection and improve biomarker discovery by reducing data dimensionality and improving classification accuracy [132]. Several recent studies have highlighted key differentially expressed miRNAs in AD. Tan et al. conducted an integrated bioinformatics and ML study to analyze blood-based miRNA expression and identified hsa-miR-6501-5p, hsa-miR-4433b-5p, and hsa-miR-143-3p as significantly upregulated in AD patients [104]. These miRNAs were linked to critical pathways, including mitochondrial dysfunction and oxidative phosphorylation, which are associated with neurodegeneration. Similarly, Liu et al. examined plasma miRNAs from the AD Neuroimaging Initiative (ADNI) and found distinct sets of miRNAs correlated with amyloid, tau, and neurodegeneration biomarker positivity [115]. Their study revealed that certain miRNAs regulate estrogen signaling receptor (ESR)-mediated signaling, which has been implicated in Aβ deposition, while others were associated with insulin-like growth factor 1 (IGF1) signaling, a key pathway in neuronal survival and neurodegeneration. Beyond blood-based studies, brain tissue analyses have also provided valuable insights into miRNA dysregulation in AD. Pang et al. performed a bioinformatic analysis of miRNAs in the entorhinal cortex and hippocampus, two regions severely affected by AD [116]. Their study identified hsa-let-7d, hsa-miR-144, hsa-miR-374a, and hsa-miR-106b as differentially expressed in AD based on the analysis of two independent datasets: GSE90828 (microarray) and GSE46579 (RNA-seq). Additionally, a recent study by Wang et al. explored human brain tissue miRNAs as a source of miRNA biomarkers [128]. Their findings indicated an exceptional decrease in miR-107 levels in AD irrespective of the stage. These results further support the hypothesis that miRNA dysregulation is not only a byproduct of AD pathology but also an active contributor to disease progression. 

### 7.2. Machine Learning Approaches to miRNA Profiling

ML algorithms can be used to build predictive models that can identify AD patients based on their miRNA expression profiles. These computational models provide a powerful framework for classifying disease states, predicting disease progression, and uncovering novel miRNA biomarkers linked to AD pathology. Supervised learning techniques, such as support vector machines (SVMs), random forests, and neural networks, have been widely applied to distinguish AD patients from healthy controls [133,134,135]. Additionally, unsupervised methods, including clustering and dimensionality reduction techniques like principal component analysis (PCA) and t-distributed stochastic neighbor embedding (t-SNE) help uncover hidden patterns within high-dimensional miRNA datasets [136]. The application of ML in miRNA-based AD prediction typically follows a structured pipeline. First, raw miRNA expression data undergo preprocessing, including normalization, batch effect correction, and feature selection to remove noise and irrelevant variables. Feature selection techniques such as the least absolute shrinkage and selection operator (LASSO) and recursive feature elimination (RFE) [132,137] are often used to retain the most informative miRNAs. Next, labeled datasets are split into training and test sets, followed by model training using various ML algorithms. Cross-validation techniques, such as k-fold cross-validation, are employed to optimize model performance and prevent overfitting. Evaluation metrics, including accuracy, precision, recall, F1-score, and area under the receiver operating characteristic curve (AUC-ROC) [138], are typically used to assess predictive accuracy. Furthermore, explainability techniques, such as SHapley Additive exPlanations (SHAP), Local Interpretable Model-Agnostic Explanations (LIMEs), and feature importance analysis, provide insights into the contribution of specific features to classification outcomes [139]. Ensemble learning approaches, such as adaptive boosting (AdaBoost) and extreme gradient boosting (XGBoost), have shown promise in improving classification accuracy [121,140]. Deep learning models, particularly deep neural networks (DNNs) and convolutional neural networks (CNNs) [117], are also being explored for capturing complex nonlinear relationships within miRNA expression data. Several studies have successfully applied ML models to predict AD and identify miRNA biomarkers. Alamro et al. developed an ML framework to analyze miRNA expression data, employing SVMs, random forests, deep neural networks (DNNs), and convolutional neural networks (CNN) to classify AD patients and ultimately identify a panel of miRNAs with high predictive value [117]. Li et al. utilized an integrative ML approach incorporating 3D genomic information, combining miRNA expression with chromatin interaction networks to highlight key miRNAs associated with AD [119]. Another study explored various ML models, including random forests and decision trees, revealing that miR-3184-5p is detrimental to normal brain function [120]. A meta-analysis incorporating AdaBoost ensemble learning demonstrated that integrating multiple datasets enhances the generalizability of ML models for miRNA-based biomarker detection [140].

### 7.3. Network Analysis Approaches to miRNA Profiling

Network analysis methods can be used to identify miRNAs involved in key pathways that are dysregulated in AD. This approach can help identify miRNAs that are potential therapeutic targets for AD. Various computational techniques, including weighted gene co-expression network analysis (WGCNA) and protein–protein interaction (PPI) networks, have been employed to elucidate the regulatory roles of miRNAs in AD pathogenesis [141,142,143]. Network-based approaches typically begin with miRNA expression profiling, followed by differential expression analysis to identify candidate miRNAs associated with AD [126,127,144]. Co-expression networks, such as WGCNA, are then constructed to group highly correlated miRNAs into distinct modules, highlighting their functional relationships [145,146,147]. PPI networks derived from STRING or BioGRID databases can further map the interactions between miRNA-regulated proteins, revealing key hubs in AD-related pathways [148,149,150]. Graph theory metrics, such as betweenness centrality and closeness, are applied to pinpoint influential miRNAs within the network [142]. Several studies have demonstrated the effectiveness of network-based approaches in identifying miRNA biomarkers and therapeutic targets for AD. Idrissi et al. constructed a PPI network, an RNA-protein interaction network, and a drug–protein interaction to identify druggable protein targets that are regulated by upregulated miRNAs in AD [122]. Another study integrated miRNA-transcription factor interactions with drug repurposing strategies, highlighting miRNAs such hsa-mir-26b-5p and hsa-mir-192-5p as critical regulators of δ-secretase—an enzyme linked to tau pathology [123]. Beyond these findings, ceRNA network analysis has uncovered key regulatory relationships between circRNAs, miRNAs, and mRNAs. Zhang et al. found that AD pathology could result from the downregulation of has_circ_002048 which causes overexpression of several miRNAs that inhibit AP2M1, a critical gene for function [124]. Additionally, blood-based miRNA expression data analyzed through WGCNA further identified several hub miRNAs such as hsa-miR-30d-5p, hsa-miR-186-5p, and hsa-miR-151b, which are dysregulated in AD [125]. Network analysis has proven to be a valuable approach for identifying key miRNAs involved in AD pathogenesis, offering insights into potential therapeutic and diagnostic biomarkers.

### 7.4. Pathway Enrichment Analysis of miRNA Pathways

Pathway enrichment analysis methods are widely used to identify biological pathways that are significantly associated with differentially expressed miRNAs in AD. These approaches help uncover the molecular mechanisms underlying AD pathology and highlight potential therapeutic targets by linking miRNAs to specific biological functions. Gene set enrichment analysis (GSEA), overrepresentation analysis (ORA), and network-based pathway analysis are common strategies employed to interpret miRNA expression data in the context of biological pathways. Pathway enrichment analysis involves several key steps. First, differentially expressed miRNAs are identified from experimental datasets, often using statistical methods such as fold-change analysis combined with false discovery rate (FDR) correction. Next, the target genes of these miRNAs are identified using databases like TargetScan [151] and miRTarBase [152]. Once the target genes are identified, pathway enrichment tools such as Kyoto Encyclopedia of Genes and Genomes (KEGG) pathway analysis [153], gene ontology (GO) term analysis [154,155], and Reactome [156] pathway mapping are employed to determine which biological pathways are significantly affected. A study by Bakulin et al. demonstrated the effectiveness of a novel computational framework for improving pathway enrichment analysis accuracy [157]. This study identified key regulators in AD by performing gene expression analysis both at tissue and cell levels—correcting for systematic biases in traditional enrichment methods. Another study by Dong et al. used pathway enrichment analysis to investigate exosomal miRNAs as potential AD biomarkers [158]. The study highlighted dysregulated pathways linked to neuroinflammation and synaptic dysfunction, reinforcing the role of miRNA-mediated regulatory mechanisms in AD progression. In the study by Qin et al., pathway enrichment analysis was applied to both mRNA and miRNA profiles to identify shared biological pathways associated with AD and MCI [159]. They implemented Mfuzz clustering and WGCNA methods, followed by the construction of a mRNA–miRNA network and pathway enrichment analysis. This study showed that miR-6734-3p and its target mRNA CYTH4 are promising biomarkers for AD.

### 7.5. Text Mining Approaches to miRNA Profiling

Text mining employs natural language processing (NLP) and ML techniques to extract meaningful insights from large volumes of scientific research. In the AD literature, text mining is particularly useful for identifying associations between miRNAs, genes, metabolites, drugs, and AD by systematically analyzing published studies, abstracts, and biomedical databases. Text mining involves several key steps, including corpus collection, text preprocessing, named entity recognition (NER), and relation extraction. NER algorithms identify mentions of miRNAs, genes, and diseases, while ML and deep learning models, such as transformer-based neural networks, extract meaningful relationships from the text [160,161,162]. In a study associating miRNAs with diseases, an automated tool, miRiaD was created to systematically extract miRNA–disease associations from the biomedical literature [163]. Additionally, a study leveraging deep learning for literature-based discovery utilized transformer-based neural networks to extract a dataset of miRNA–disease relations, demonstrating the potential of deep learning in text mining for AD research [164]. Expanding on these efforts, transformer-based models have been further refined for miRNA–disease association predictions. For instance, MDformer integrates multi-source biological features with transformer architecture to enhance the prediction of miRNA–disease links, demonstrating improved accuracy over traditional models [165]. Similarly, the Dual-Channel Transformer Graph Model (DCTGM) employs a combination of graph-based learning and transformer networks to capture complex associations between miRNAs and diseases, further advancing automated biomedical knowledge extraction [166]. Given the growing success of these transformer-based models in miRNA–disease association studies, applying similar approaches to AD research could provide valuable insights. AD is characterized by complex genetic and molecular interactions and leveraging state-of-the-art text mining techniques could help uncover novel miRNA biomarkers, disease mechanisms, and potential therapeutic targets. By adapting these transformer-based frameworks to AD-focused datasets, researchers could systematically extract and predict novel associations, contributing to a broader understanding of AD pathogenesis and treatment strategies.

## 8. miRNAs as Biomarkers in FTD

Dysregulated miRNAs have also been identified in the blood and brain of FTD patients, with the possibility of using these miRNAs for early diagnosis being crucial, given the difficulty of diagnosing FTD due to its heterogeneous clinical presentation. However, inconsistencies across studies limit the full potential of miRNAs in FTD diagnostics. Blood remains the most attractive matrix for biomarker discovery due to its accessibility and noninvasive nature. Sheinerman et al. investigated the levels of preselected miRNAs in blood plasma from healthy controls and FTD, AD, PD, and amyotrophic lateral sclerosis (ALS) patients, aiming to identify miRNA pairs and combinations that could accurately discriminate each disease from controls [167]. The selected miRNAs were mostly those associated with synapses, brain regions affected by the target pathologies, or linked to inflammatory processes and apoptosis. miR-9-3p/let-7e, miR-7/miR-451, and miR-335-5p/let-7e pairs performed best in distinguishing FTD from controls (AUC of 0.79, 0.75, and 0.83, respectively), with their combination yielding an AUC of 0.94, sensitivity of 0.86, and specificity of 0.90. In distinguishing FTD from AD, miR-125b/miR-29a, miR-125b/miR-874, and miR-107/miR-335-5p pairs showed the best performance (AUC of 0.78, 0.75, and 0.80, respectively), and their combination had an AUC of 0.87, sensitivity of 0.78, and specificity of 0.76. To distinguish FTD from ALS, miR-129-3p/miR-206 and miR-338-3p/let-7e pairs performed best (AUC of 0.86 and 0.81, respectively), with their combination achieving an AUC of 0.94, sensitivity of 0.84, and specificity of 0.86. Additionally, miR-335/let-7e and miR-99b/let-7e classifiers and miR-9-3p/miR-181a performed best in distinguishing FTD from controls across all participants (AUC of 0.94). Denk et al. examined miRNA expression levels in blood serum from patients with behavioral variant FTD (bvFTD), patients with AD, and healthy controls [168]. They found that bvFTD patients had significantly higher expression levels of miR-103a-3p, miR-142-3p, miR-20a-5p, miR-29b-3p, miR-143-3p, miR-197-3p, miR-27a-3p, miR-338-3p, miR-491-5p, miR-7b-5p, miR-7g-5p, miR-106a-5p, miR-106b-5p, miR-18b-5p, miR-223-3p, miR-26a-5p, miR-26b-5p, miR-301a-3p, and miR-30b-5p, while lower expression levels of miR-132-3p, miR-100-5p, miR-335-5p, miR-99a-5p, miR-146a-5p, miR-15a-5p, miR-22-3p, miR-320a, miR-320b, miR-92a-3p, and miR-1246 were observed compared to controls. Grasso et al. identified three miRNAs—miR-663a, miR-502-3p, and miR-206—in the plasma of FTD patients that were significantly downregulated compared to healthy controls (HCs) [169]. These miRNAs are involved in neurological pathways and demonstrated excellent diagnostic performance in males, with 100% sensitivity and 87.5% specificity. However, in females, their sensitivity and specificity were lower (77% and 75%, respectively). In another study, Kmetsch et al. examined plasma miRNA expression in patients with *C9orf72* mutations and presymptomatic carriers [170]. They found four potential miRNA biomarkers with differential expression patterns that evolved as the disease progressed. miR-345-5p was overexpressed in patients compared to healthy controls but not in presymptomatic carriers, suggesting its involvement in disease progression. miR-34a-5p was overexpressed in patients and presymptomatic carriers, indicating deregulation associated with the *C9orf72* mutation. miR-200c-3p and miR-10a-3p were underexpressed in patients, suggesting that their deregulation occurs only after the disease is fully established. These four miRNAs could distinguish presymptomatic carriers and patients from healthy controls (AUC of 0.90 for both) and patients from presymptomatic carriers (AUC of 0.80). Magen et al. profiled blood plasma miRNAs in a separate study and developed a miRNA-based classifier for diagnosing FTD [171]. This classifier was trained using a cohort of 219 subjects and validated in a separate cohort of 74 subjects. The classifier, based on 13 miRNAs (miR-629-5p, miR-361-5p, miR-125b-5p, miR-326, miR-484, miR-26a-5p, miR-423-5p, miR-7c-5p, miR-107, miR-185-5p, miR-379-5p, miR-378a-3p, and miR-7d-3p), successfully distinguished FTD from non-neurodegenerative controls in approximately 90% of cases. The miRNAs that contributed most to the classification included miR-629 (associated with multiple sclerosis), miR-125b (brain-enriched), and miR-361 (astrocyte-derived exosomal). Within plasma-derived EVs, miR-181c, which may mediate its effects through microglial-directed neuroinflammation and interaction with TAR DNA-binding protein 43 (TDP-43), was found to be significantly downregulated in patients with FTD compared to healthy controls [172]. Moreover, the analysis of plasma-derived NDEVs revealed that miR-92a-3p and miR-320a levels were significantly upregulated in FTD patients compared to healthy controls and AD patients [13]. However, identifying miRNAs as biomarkers for FTD presents a challenge due to the overlap with other disorders, particularly those that share genetic variants or pathological features with FTD. Differentially expressed miRNAs specific to a particular genetic mutation may not be informative for other genetic variants or sporadic cases and may not effectively differentiate FTD from other overlapping conditions [173]. In conclusion, these findings (Table 3) emphasize the need for further research to identify blood-based miRNA signatures that can differentiate between healthy individuals and patients with AD and FTD, potentially even in the prodromal stage. Such signatures, combined with other blood-based biomarkers and genetic predictors, could significantly aid in risk stratification for large-scale prevention programs, early therapeutic interventions, disease stage assessment, and the monitoring of disease progression or therapeutic effects. Although studies have not explored the presence of oxidized miRNAs in circulation, 8-oxo-7,8-dihydroguanosine has been detected in bodily fluids, including urine and blood, and suggested as a biomarker for metabolic conditions [174,175]. Further research is warranted to explore the potential of oxidized miRNAs as biomarkers for AD and dementia.

## 9. Computational Approaches to Discover miRNA Biomarkers for FTD

The computational approaches discussed in Section 7 for AD apply to FTD as well. Several studies encompass differential expression analysis, ML, network analysis, pathway enrichment analysis of miRNA pathways, and text mining approaches in miRNA profiling for FTD. For example, Kmetzsch et al. (2020) analyzed plasma samples from individuals with *C9orf72* mutations and identified a signature of four miRNAs that were differentially expressed across clinical conditions: miR-34a-5p and miR-345-5p (upregulated) and miR-200c-3p and miR-10a-3p (downregulated). This signature shows promise as a biomarker for progression in C9orf72-associated FTD and ALS [170]. In a subsequent study in 2022, Kmetzsch and colleagues examined circulating miRNA signatures in both symptomatic and presymptomatic carriers of *MAPT* and *GRN* mutations. They identified several miRNAs that were differentially expressed in each cohort, indicating distinct miRNA profiles for each FTD form [173] (Table 4). Additionally, Piscopo et al. investigated the differential expression of nine circulating miRNAs linked to apoptosis and compared them with AD patients. They found that the plasma levels of miR-127-3p were significantly lower in FTD patients than in those with AD, suggesting that miR-127-3p could serve as a biomarker to differentiate FTD from AD. These studies highlight the potential of miRNA profiling as a tool for distinguishing FTD from other neurodegenerative diseases and identifying specific miRNA signatures associated with various genetic mutations linked to FTD [176]. In terms of applying ML approaches to identify miRNA biomarkers for FTD, Magen et al. developed a nonlinear prediction model using cell-free plasma miRNA profiles to distinguish FTD patients from non-neurodegenerative controls. Their model achieved about 90% accuracy after narrowing the list down to 13 miRNAs and including gender and age as features, underscoring the potential of miRNA biomarkers for early-stage FTD detection [171]. This study demonstrates the efficacy of ML methodologies in analyzing miRNA expression data and discovering reliable biomarkers for FTD diagnosis and progression monitoring. Regarding network approaches, Swarup et al. applied weighted gene co-expression network analysis (WGCNA) to miRNA data focused on the frontal and temporal cortices—regions typically affected in FTD. Their analysis identified miR-203 as an overexpressed hub within a regulatory miRNA module, mirroring mRNA co-expression patterns linked to disease progression. miR-203 was found to promote neuronal cell death, suggesting its role as a key regulator of neurodegeneration and a conserved gene network implicated in FTD pathogenesis [177]. Though the application of network analysis, such as WGCNA, in miRNA research related to FTD is still evolving, several studies, including Ferrari et al., have applied WGCNA to explore gene expression patterns in neurodegenerative diseases. They identified genes relevant to FTD pathology, including *MAPT*, *GRN*, *CHMP2B*, and others, by performing co-expression analysis of genes in the frontal and temporal cortices [178]. Pathway enrichment analysis of miRNA data has proven essential in uncovering the molecular mechanisms behind FTD. For instance, Schneider et al. investigated the downregulation of exosomal miR-204-5p and miR-632 in FTD patients. Pathway enrichment analysis revealed that these miRNAs are involved in synaptic function and neuronal communication pathways, such as Wnt signaling, MAPK signaling, and neurotrophin signaling, suggesting their potential role in FTD pathogenesis [179]. Kaurani et al. focused on astrocytic miR-129-5p and demonstrated its involvement in neuroinflammatory pathways relevant to FTD. Their pathway analysis indicated that miR-129-5p targets genes associated with neuroinflammation, highlighting its potential as a therapeutic target [180]. These studies emphasize the importance of pathway enrichment analysis in identifying key miRNA-mediated pathways involved in FTD, offering potential biomarkers and therapeutic targets. Overall, computational approaches such as these are invaluable for exploring the role of miRNAs as blood biomarkers in FTD.

## 10. General Challenges and Future Directions with miRNAs in Dementia Research

Despite the progress that has been made in the computational identification of miRNA biomarkers for AD and FTD, there are still several challenges that need to be addressed. These include the following:

- Heterogeneity of phenotypes: All forms of dementia are complex and heterogeneous diseases, and miRNA expression profiles can vary significantly between individuals. This makes it challenging to identify miRNA biomarkers that are consistently accurate across different populations.
- Heterogeneity of the miRNA population: While some miRNAs are brain-enriched and primarily reflect neurodegenerative processes, others can be secreted from various peripheral tissues and organs. Moreover, systemic conditions, such as inflammation or metabolic disorders, can influence the levels of circulating miRNAs and may act as confounding factors, potentially affecting their specificity as biomarkers for neurodegenerative diseases.
- Lack of standardized protocols: There is a lack of standardized protocols for miRNA extraction, quantification, and analysis. This can lead to a high degree of variability in results between different studies.
- Validation in large cohorts: Many studies on miRNA biomarkers for AD have been conducted on small cohorts. Larger studies are needed to validate the findings of these studies and to confirm the clinical utility of miRNA biomarkers.

Future research in this field should focus on addressing these challenges and developing more robust and accurate computational methods for miRNA-based biomarker discovery. This will help pave the way for the development of new diagnostic and therapeutic strategies for AD and FTD.

### Methodological Challenges to the Use of miRNAs as Biomarkers

The easy and non-invasive accessibility of miRNAs in body fluids, their long half-life, and their therapeutic potential have positioned circulating miRNAs as a key focus in translational research. However, while an increasing number of studies report differential miRNA expression in relation to disease states, most lack procedural uniformity, especially standardized protocols, making it challenging to compare or reproduce results across studies. For this reason, a clear understanding of the strengths and weaknesses of sampling, analysis methods, and the context of use is critical before translating research into clinical practice [181]. Several factors complicate the interpretation of circulating miRNA levels, including age, gender, ethnicity, drug use, and smoking [182]. Studies on mouse serum suggest that miRNA concentrations in blood may vary according to circadian rhythms, although these variations have not been confirmed in humans [183]. Variability in the assessment of miRNAs from body fluids also stems from differences in extraction methodologies and analytical methods, which can lead to inconsistent results. In blood, miRNAs are either bound to proteins or encapsulated in EVs. While these associations contribute to miRNA stability, they can also complicate the extraction process and lead to different outcomes. RNA and miRNA extraction methods generally rely on either phenol–chloroform-based organic extraction or a combination of organic extraction followed by selective solid-phase purification onto mini-columns, with subsequent elution in water or a buffer [184]. The choice of extraction method is crucial and should consider the matrix from which the RNA is being extracted. For example, the combined organic and column-based extraction method has been shown to yield better results for small RNAs from exosomes compared to phenol-only extraction [185]. The methods used for miRNA expression profiling also present potential pitfalls. Common platforms include qRT-PCR, droplet digital PCR (ddPCR), microarrays, and miRNA sequencing (miRNA-seq). Each method has its own advantages and limitations, which can result in inconsistent profiling outcomes when compared. For instance, qRT-PCR is often more sensitive than microarrays for profiling miRNAs from body fluids [186]. However, qRT-PCR is limited to a small number of miRNAs, while microarrays and next-generation sequencing (NGS) can profile a wider range. Moreover, RT-qPCR should not be performed on samples collected in heparin-coated blood collection tubes, as heparin inhibits reverse transcriptases and polymerases, reducing RNA yield [187]. Another challenge with RT-qPCR is the choice between absolute and relative quantification methods. Absolute quantification, which measures the number of copies of a target gene using a standard curve, is impractical for profiling large numbers of miRNAs. Relative quantification, which compares the miRNA of interest to an assumed invariant reference miRNA, can be problematic due to the lack of a universally stable endogenous control [181]. To address this, researchers often add exogenous calibrators or use mean normalization with endogenous miRNAs that are stable across samples [188]. Microarrays, in contrast to qPCR, are hybridization-based detection methods that offer more comprehensive coverage and, in some cases, can be customized to meet specific research needs [129,189]. Lastly, NGS appears to be the most promising technology for miRNA analysis. Unlike other methods, NGS does not require prior knowledge of target miRNAs or the use of specific probes or primers, and oxo-G was demonstrated to be possible to detect using o8G-Seq, allowing the profiling of oxidized miRNAs [73]. This opens the door to discovering novel small RNAs and allows for more comprehensive studies beyond known miRNAs. To conclude, despite the great potential of miRNAs as non-invasive and informative biomarkers, several challenges remain. These include biases associated with sample type, collection, storage, and purification, as well as the absence of standardized quantification protocols. Additionally, there is still a lack of understanding of their physiological variation in biofluids, which complicates their application as reliable biomarkers.

## 11. Conclusions

In summary, there are currently no disease-modifying treatments that can halt the progression of AD or FTD. The success of new therapeutic trials largely depends on advancements in biomarker research. Among potential noninvasive biomarkers for neurodegenerative diseases, circulating miRNAs present a promising approach. Significant progress has been made in identifying miRNAs in blood or encapsulated within EVs as easily accessible biomarkers that distinguish AD and FTD patients from healthy controls and those with other neurodegenerative disorders with overlapping symptoms. The exact role of miRNAs in AD and FTD, particularly in disease onset and progression, is yet to be fully understood and research is ongoing. However, mounting evidence suggests that miRNAs may play both causal and secondary roles in these conditions. Indeed, miRNAs regulate the expression of genes crucial for neuronal function, survival, and plasticity, as well as disease-related pathways, such as Aβ production, tau phosphorylation, and synaptic function. Simultaneously, as neurodegeneration progresses, cellular stress, inflammation, and neuronal injury can lead to altered miRNA expression, potentially reflecting the body’s response to the disease. These changes may also be part of a compensatory mechanism, where the body adjusts gene expression in an attempt to protect neurons or mitigate inflammation. Given the multifaceted roles of miRNAs in biological systems, it remains challenging to definitively distinguish whether these molecules are directly causal or simply reflective of the secondary effects of neurodegeneration. Despite these challenges, ongoing research holds promise for clarifying the role of miRNAs in neurodegeneration. With further advancements, measuring miRNAs in biofluids could eventually become a minimally invasive, sensitive, and specific diagnostic tool for monitoring and diagnosing neurodegenerative diseases. This could be used alongside clinical practice, neuroimaging, genetic markers, and other diagnostic tools. Furthermore, given their role in regulating key pathogenic processes, identifying disease-specific miRNAs could open new avenues for developing targeted therapeutic interventions. However, to truly harness the potential of miRNAs as diagnostic and prognostic biomarkers, standardization of both preanalytical and analytical factors is crucial and still needs to be addressed. 

## Figures and Tables

**Table 1:** miRNAs deregulated in AD.

miRNA	Direction of Changes	Biological Effects	Biological Specimen/Animal Models	References
miR-149	↓	Target BACE1 to modulate Aβ production	SH-SY5Y	[89]
miR-34a-5p	↓		MCN, N2a	[88]
miR-125b-5p	↓			
miR-15b	↓		SH-SY5Y	[87]
miR-16	↓		AD brain, PC12, N2a	[86]
miR-124	↓		SH-SY5Y	[85]
miR-374b-5p	↓		SH-SY5Y, BV2	[84]
miR-125b	↑	Promote tau phosphorylation	Rat hippocampal and cortical neurons	[91]
miR-483-5p	↑		HEK293	[93]
miR-16-5p	↑	Promote apoptosis via BCL-2	5xFAD mice, SH-SY5Y	[97]
miR-34a	↑	Modulate synaptic plasticity	AD brain, 3xTg-AD mice	[99]
miR-92a-3p	↑		Plasma samples	[101]
miR-181c-5p	↑			
miR-210-3p	↑			
miR-146b-5p	↓	Modulate innate immune system and cytokine pathways	Whole blood	[102]
miR-15b-5p	↓	Target cell cycle and apoptosis		
miR-6501-5p	↓	Modulate oxidative phosphorylation, mitochondrial dysfunction, and calcium-mediated signaling	Whole blood	[104]
miR-4433b-5p	↓			
miR-143-3p	↓			
miR-26b	↓	Associated with the immune system, cell cycle, gene expression, cellular response to stress, neuron growth factor signaling, Wnt signaling, cellular senescence, and Rho GTPases	Whole blood	[108]
miR-30e	↑		Exosomes and serum samples	
miR-34a, miR-107	↓		Plasma samples	
miR-200c	↑			
miR-34c	↑		Serum samples	
miR-125b, miR-210, miR-485	↓			
miR-146a	↓		Plasma and serum samples	
miR-30e-5p, miR-18b-5p, miR-424-5p, miR-582-5p, miR-335-5p, miR-20a-5p, miR-106a-5p, miR-361-5p, miR-15a-5p	↑	Associated with apoptosis, immune response, and inflammation	Whole blood	[109]
miR-29b-3p, miR-27b-3p, miR-221-3p, miR-146a-5p, miR-15b-3p, miR-31-5p, miR-9-5p, miR-107, miR-103a-3p, miR-1306-5p	↓		Whole blood	
miR-451a, miR-21-5p, miR-23a-3p, miR-126-3p, let-7i-5p, miR-151a-3p	↓	Implicated in metabolic processes and protein phosphorylation	Plasma-derived EVs	[110]
miR-424-5p, miR-93-5p, miR-3065-5p	↑	N/A	Plasma-derived EVs	[111]
miR-1306-5p	↓			
miR-106a-5p, miR-16-5p, miR-17-5p, miR-195-5p, miR-19b-3p, miR-20a-5p, miR-223-3p, miR-25-3p, miR-296-5p, miR-30b-5p, miR-532-3p, miR-92a-3p, miR-451a	↑	Regulate the TGF-β signaling pathway and apoptosis and suppress APP	Plasma-derived EVs	[112]
miR-23a-3p, miR-223-3p, miR-190a-5p	↑	Regulate axon guidance, long term depression, and calcium signaling	Plasma-derived EVs	[113]
miR-100-3p	↓			
miR-106b-5p, miR-107, miR-135b-5p	↑	Target BACE1, promote neuronal damage (inducing hypoxia and M1-type microglia polarization), regulate cytokine and chemokine release from microglia, and control BBB integrity	Plasma-derived EVs	[114]
miR-29a-5p, miR-9-5p, miR-125b-5p, miR-132-5p, miR-210-3p	↑↓			

BACE1, β-site APP-cleaving enzyme 1; BCL-2, B-cell lymphoma 2 protein; Rho GTPases, Rho family of GTPases; TGF-β, transforming growth factor β; APP, amyloid precursor protein; BBB, blood–brain barrier; SH-SY5Y, neuroblastoma cell line; MCN, primary mouse cortical neurons; AD, Alzheimer’s disease; PC12, cellular AD model; N2a, Neuro2a; BV2, murine microglial cell line; HEK293, human embryonic kidney 293 cells; 5xFAD, transgenic mouse model that rapidly develops severe amyloid pathology; 3xTg-AD, transgenic mouse model that contains three mutations associated with familial AD (APP Swedish, MAPT P301L, and PSEN1 M146V); EVs, extracellular vesicles; N/A, not available; ↑, upregulated; ↓, downregulated, ↑↓, either up- or downregulated.

**Table 2:** Summary of computational approaches and the discovered miRNA biomarkers in AD.

miRNA	Methods	References
hsa-miR-6501-5p, hsa-miR-1296-5p, hsa-miR-1307-3p, hsa-miR-4433b-5p, and hsa-miR-143-3p	TMM normalization, differential expression with EdgeR 3.38.4, hybrid carss-SVMRFE feature selection, miRNA target prediction (DIANA-microT-CDS v5.0), KEGG pathway analysis (DIANA-miRPath v3.0), and network visualization (Cytoscape version 3.9.1 Bioinformatics Resources 6.7).	[104]
hsa-miR-483-3p, hsa-miR-145-5p, hsa-miR-374a-3p, hsa-miR-1180-3p, hsa-miR-337-5p, and hsa-miR-1224-5p, hsa-miR-652-3p, hsa-miR-95-3p, hsa-miR-339-5p, hsa-miR-628-5p, hsa-miR-190a-5p, and hsa-miR-3679-5p, hsa-miR-1255b-5p, hsa-miR-941, hsa-miR-369-5p, hsa-miR-193b-5p, hsa-miR-215-5p	miRNA expression analysis (DESeq2 version 1.42.1), target prediction (mirTarBase Release 9.0), network analysis with STRING database and Cytoscape version 3.10.1, pathway analysis (Metascape v3.5.20240101), disease classification (STREAMLINE, release beta 0.3.4), and feature importance via mutual information and MultiSURF algorithm.	[115]
has-let-7d, has-miR-144, has-miR-374a, has-miR-106b	WGCNA for gene coexpression, GO/KEGG analysis (DAVID Bioinformatics Resources 6.7), gene comparison (FunRich 2.1.2.), drug screening with CMAP (Available from: https://www.broadinstitute.org/connectivity-map-cmap, accessed 17 February 2025), and miRNA differential expression (Limma 3.28.14 R Package).	[116]
hsa-mir-16-5p, hsa-mir-34a-5p, hsa-mir-1-3p, hsa-mir-26a-5p, hsa-mir-93-5p, hsa-mir-155-5p	Meta-analysis of GEO datasets with ImaGEO2 (Available from: https://imageo.genyo.es/, accessed 17 February 2025), network analysis (STRING database 2021, Cytoscape version 3.9.1), feature selection (LASSO), classification (RF, SVM, DNN, CNN algorithms), and gene set analysis using DisGeNET in Enrichr (Available from: https://maayanlab.cloud/Enrichr/, accessed 18 February 2025).	[117]
let-7e, miR-96, and miR-484, miR-99b, miR-100, miR-30e, miR-378i, miR-145, miR-378c, miR-451a	Diff. expr. with Limma (R v3.5.1), up- and downregulation with multiMiR (R v3.5.1), target gene and KEGG pathway analysis with DIANA-miRPath 3.03.0, and GO enrichment analyses with FunRich (Available from: http://www.funrich.org/, accessed 18 February 2025).	[118]
miR-128, miR-210	miRNA name mapping (miEAA 2.0, miRBase V22), sequence alignment (bioMart 2.52.0), 3D genome modeling (Hi-C data), clustering (DBSCAN algorithm), correlation analysis (Spearman), and model training (sklearn, leave-one-out cross-validation).	[119]
hsa-miR-3184-5p, hsa-miR-1227-5p, hsa-miR-3181, hsa-miR-6088	miRNA expression profiling (GEO Available from: https://www.ncbi.nlm.nih.gov/geo/query/acc.cgi?acc=GSE120584 accessed 17 February 2025), feature selection (Boruta, mRMR, MCFS algorithms), classification models (RF, PART algorithms), incremental feature selection (IFS algorithm), oversampling (SMOTE algorithm), and performance evaluation (MCC, accuracy metrices).	[120]
let-7d-5p, miR-106b-3p, miR-107, miR-126-5p, miR-148b-5p, miR-181c-3p, miR-191-5p, miR-200a-3p, miR-22-3p, miR-483-5p, miR-486-5p, miR-502-3p, miR-93-5p	Meta-analysis (metafor 4.0.0), miRNA standardization (miRNAmeConverter 3.20, miRBase 22.1), miRNA–disease association identification (ABMDA method), disease similarity (DOSE 3.30.0), miRNA similarity (MISIM database v2.0), pathway enrichment (clusterProfiler 4.0.5, KEGG database), gene network analysis with STRING (version 11), target gene retrieval (multiMiR release 3.12, miRTarBase 9.0), and risk of bias assessment (MIQE).	[121]
hsa-miR-26a-5p, hsa-miR-107, hsa-miR-26b-5p or hsa-let-7f-5p	Protein collection (pubmed2ensembl v1.0, UniProtKB v1.0), gene set enrichment (ClueGo v1.0, CluePedia v1.0, GO database, Reactome database), protein interaction network (STRING version 11, CentiScaPe v1.0), miRNA and lncRNA targeting (miRTarBase Homo sapiens 8.0 database, LncRNA2Target 2.0 database, CyTargetLinker v4.1.0), drug–gene interaction (Drug–Gene Interaction database 3.0, Cytoscape 3.7.2).	[122]
hsa-mir-26b-5p, hsa-mir-192-5p, hsa-let-7e-5p, hsa-let-7f-5p, hsa-mir-124-3p, hsa-mir-20a-3p, hsa-mir-217, hsa-mir-433-3p, hsa-mir-1-3p, hsa-mir-128-3p, hsa-mir-129-2-3p, hsa-mir-146a-5p, hsa-mir-194-5p, hsa-mir-23b-3p, hsa-mir-34a-5p, hsa-mir-375	PPI network construction (Genemania database 2008, Cytoscape, Network Analyzer), miRNA identification (Starbase v2.0, miRWalk v3.0, miRanda v1.0), TF identification (Network Analyst 3.0, TRRUST v2.0, ENCODE registry v2), expression validation (GTEx v8, Brainspan – Available from https://www.brainspan.org/, accessed 17 February 2025; BrainEXP release 2019), shape-based screening (Pubchem, Swiss Similarity, CHEMBL database – Available from https://www.ebi.ac.uk/chembl/, accessed 17 February 2025), ligand–protein preparation (Schrödinger 14-2, Ligprep Schrödinger module), docking (Glide release 2021), molecular dynamics (Desmond), and normal mode analysis (iMODS server release 2014).	[123]
hsa-miR-422a, hsa-miR-4784, hsa-miR-3944-3p	Gene expression data mapping (GEO database – Available from https://www.ncbi.nlm.nih.gov/geo/, accessed 17 February 2025, miRNA: miRBase V22), data processing (GEO2R– Available from https://www.ncbi.nlm.nih.gov/geo/geo2r/, accessed 17 Febraury 2025, R: limma & clusterProfiler from Bioconductor release 3.15, GGplot2 3.3.6, enrichPlot in Bioconductor 3.15, survival 3.3-13.3-1), pathway analysis (DAVID v2022q3, KEGG release 104.0, GO database), PPI network construction (STRING database 11.5, Cytoscape 3.9, CytoHubba, Between Centrality), survival analysis (R: survival, Cox method, Kaplan–Meier), ceRNA network analysis (Cytoscape 3.9, ENCORE database 2022), and clinical validation (GSEA, correlation analysis with MMSE, NFT, Braak).	[124]
hsa-miR-30d-5p, hsa-miR-186-5p, hsa-miR-425-5p, hsa-miR-4781-3p, hsa-miR-361-5p, hsa-miR-26b-3p, hsa-miR-30a-5p, hsa-miR-30a-3p, hsa-miR-30e-3p, hsa-miR-151a-5p, hsa-miR-151b	Data collection (GEO, GSE46579, GSE85426), preprocessing (quantile normalization), differential expression (limma, FDR < 0.01), WGCNA, miRNA–mRNA network (miRWalk 2.0, Cytoscape 3.7.2), RT-PCR validation, and statistical analysis.	[125]
miR 26b-5p, miR-103a-3p, miR-107, miR-26a-5p, let-7f-5p, miR 532-5p, miR-151a-3p, miR-5010-3p, miR-1285-5p, let-7d-3p	Data preprocessing (background correction, Limma package), differential expression (Student’s t-test, Stouffer test, FDR < 0.001), miRNA–target prediction (RNA22 v2.0, miRanda v1.0, miRDB v5.0, miRWalk v1, PICTAR2 v1, TargetScan 7.1), functional annotation using GeneCodis (Available from: https://genecodis.genyo.es/, accessed 17 February 2025), GO and KEGG databases, miRNA–target network with Cytoscape 3.5.1, and RT-qPCR validation.	[126]
miR-30a-5p, miR-335	miRNA–gene interaction network (Cytoscape 3.2.1, ClueGO v2.2.5), microarray data preprocessing (affy, plier, piano packages in R), differential expression using GEO2R tool (Available from: https://www.ncbi.nlm.nih.gov/geo/geo2r/, accessed 18 February 2025), RNA-seq data analysis (FastQC v0.11.5, TopHat2 version 2.0.8, Cuffdiff v1), miRNA–target gene identification with cyTargetLinker ( Available from: https://apps.cytoscape.org/apps/cytargetlinker, accessed 18 February 2025) using miRTarBase, TarBase v.8 and miRWalk 2.0 databases, gene ontology and pathway analysis (ClueGO v2.2.5), and clustering (gplots, RcolorBrewer1.1-2).	[127]

miRNA, microRNA; lncRNA, long non-coding RNA; WGCNA, weighted gene co-expression network analysis; PPI, protein–protein interaction; KEGG, Kyoto Encyclopedia of Genes and Genomes; GO, gene ontology; TMM, Trimmed Mean of M-values; SVM-RFE, support vector machine-based recursive feature elimination; GEO, Gene Expression Omnibus; ceRNA, competing endogenous RNAs.

**Table 3:** miRNAs involved in FTD.

miRNA	Direction of Changes	Biological Effects	Biological Specimen/Animal Models	References
miR-103a-3p, miR-142-3p, miR-20a-5p, miR-29b-3p, miR-143-3p, miR-197-3p, miR-27a-3p, miR-338-3p, miR-491-5p, miR-7b-5p, miR-7g-5p, miR-106a-5p, miR-106b-5p, miR-18b-5p, miR-223-3p, miR-26a-5p, miR-26b-5p, miR-301a-3p, miR-30b-5p	↑	N/A	Serum samples	[168]
miR-132-3p, miR-100-5p, miR-335-5p, miR-99a-5p, miR-146a-5p, miR-15a-5p, miR-22-3p, miR-320a, miR-320b, miR-92a-3p, miR-1246	↓			
miR-663a, miR-502-3p, miR-206	↓	Modulate inflammatory responses, cell proliferation, and BDNF protein synthesis	Plasma samples	[169]
miR-345-5p, miR-34a-5p	↑	Involved in cell signaling, apoptosis, and intermediary metabolism	Plasma samples	[170]
miR-200c-3p, miR-10a-3	↓			
miR-181c	↓	Promote microglial-directed neuroinflammation and translocation of TDP-43 from the nucleus to the cytoplasm	Plasma-derived EVs	[172]
miR-92a-3p, miR-320a	↑	Regulate Tau expression, TGF-β signaling pathway, and apoptosis	Plasma-derived EVs	[13]

EVs, extracellular vesicles; BDNF, brain-derived neurotrophic factor; TDP-43, transactive response DNA binding protein 43 kDa; TGF-β, transforming growth factor β; N/A, not available; ↑, upregulated; ↓, downregulated.

**Table 4:** Summary of computational approaches and the discovered miRNA biomarkers in FTD.

miRNA	Methods	References
miR-34a-5p, miR-345-5p, miR-200c-3p, miR-10a-3p	miRNA extraction (miRNeasy Serum/Plasma Kit (Qiagen)), sequencing (Illumina NovaSeq6000), raw reads processing with FastQC (Available from: http://www.bioinformatics.babraham.ac.uk/projects/fastqc, accessed 18 February 2025), UMI-tools, Cutadapt, Bowtie, Samtools, miRDeep2), statistical analysis using EdgeR (R V.3.6.1), logistic regression (scikit-learn), cross-validation, feature selection, and pathway analysis (DIANA-miRPath V.3).	[170]
miR-34a-5p, miR-338-3p, miR-142-3p, miR-320a, miR-145-5p, miR-92a-3p, let-7 g-5p, miR-199a-5p, miR-206, miR-30b-5p, miR-191-5p, miR-27a, miR-320b, miR-143-3p, miR-1246, miR-223-3p, miR-144-3p, miR-451, miR-194-5p, miR-144-5p, miR-29b-3p, miR-29c-3p, miR-192-5p, miR-19a-3p, miR-502-3p, miR-15a-5p, miR-374b-5p, miR-7-1-3p, miR-320c, miR-106b-5p, miR-146a-5p, miR-133b, let-7b-5p, miR-345-5p, miR-22-3p	Quality control with FastQC, sequence cleaning with UMI-tools and Cutadapt, sequence alignment with Bowtie, PCR duplicate removal with UMI-tools, data quantification with Samtools idxstats v1.15, differential expression analysis with EdgeR from Bioconductor 3.14, binary classification with logistic regression, cross-validation, and ROC-AUC evaluation.	[173]
miR-127-3p	T-test or Mann–Whitney test, ROC curve analysis, sensitivity, and specificity. All statistical analyses were performed using STATA 13.	[176]
miR-423-5p, miR-125b-5p, miR-26a-5p, miR-326, miR-185-5p, miR-629-5p, miR-484, let-7d-3p, miR-107, let-7c-5p, miR-361-5p, miR-379-5p, miR-378a-5p	Classification with logistic regression, evaluation using ROC-AUC, feature selection via RFE, and feature importance analysis with SHapley Additive exPlanations (SHAP v0.44.0).	[171]
miR-203	Log2-transformed FPKM values were quantile-normalized (betweenLaneNormalization) using the EDAseq package in R (freely available at https://bioconductor.org/packages/release/bioc/html/EDASeq.html accessed 18 February 2025). PCA, WGCNA package in R (CRAN v1.68), module preservation analysis, enrichment analysis (GO-Elite), and PPI network analysis (igraph package in R v1.2.3) were performed.	[177]
miR-204-5p, miR-632	miRNA expression processing was performed prior to statistical analysis using GenEx 6 (MultiD Analyses). Statistical analysis involved a t-test with Holm–Sidak correction, logistic regression, ROC calculations, and cross-validation, all conducted using GraphPad Prism V.7.01.	[179]
miR-129-5p	Batch effect correction was performed using the RUVSeq package (freely available at https://bioconductor.org/packages/release/bioc/html/RUVSeq.html accessed 18 February 2025), followed by differential expression analysis with DESeq2. Target annotation was performed with miRTarBase v7.0, and target gene selection was based on the GTEx portal(Available from: https://gtexportal.org/home/, accessed 18 February 2025). Network analysis and pathway enrichment were conducted using tools such as NPInter v5.0, RegNetwork, Rise2.14, STRING 12.0, TarBase v9, and TransmiR v3. Bioconductor version 3.20 used.	[180]

miRNA, microRNA; ROC, receiver operating characteristic; PCR, polymerase chain reaction; RFE, recursive feature elimination; PCA, principal component analysis; WGCNA, weighted gene co-expression network analysis; PPI, protein–protein interaction.
